# Optical (Cherenkov) dosimetry to unravel the radiobiological effects of radioisotope based therapy

**DOI:** 10.1007/s00259-024-07047-4

**Published:** 2025-01-22

**Authors:** Fijs W.B. Van Leeuwen, Jean-Pierre Pouget, Michael Lassmann

**Affiliations:** 1 Interventional Molecular Imaging laboratory, Leiden University Medical Center, Leiden, The Netherlands; 2 Institut de Recherche en Cancérologie de Montpellier (IRCM), Institut National de la Santé et de la Recherche Médicale (INSERM) Unité 1194, Université de Montpellier, Institut Régional du Cancer de Montpellier (ICM), Montpellier, France; 3 Department of Nuclear Medicine, University Hospital Würzburg, Würzburg, Germany

Optical luminescence imaging provides possibilities to extend the field of molecular imaging beyond nuclear medicine. Due to the relatively high resolution and the widespread availability, the use of optical imaging is common in biomedical studies (cells and small animals based). Unfortunately, the severe tissue attenuation of light limits clinical uses of luminescnece-based optical techniques to superficial applications in ophthalmology (the eye is transparent), (endoscopic) surgery (during the surgical excisions targets appear at the surface), dermal analysis, tissue surface, and via imaging. Optical technologies come in many forms. When a charged particle (e.g. a β^+^/β^−^-emission) moves through a dielectric medium at a speed greater than the speed of light in that medium it can induce the emission of light, which is called Cherenkov luminescence (CL; continuous spectrum with emission max in the UV) [1, 2]. CL is representative for the interaction between an ionizing radiation and the medium it travels in. Most well-known uses of CL in nuclear medicine exploit PET radiopharmaceuticals for optical guidance towards superficial lesions [3].

Being able to deliver the right absorbed radiation dose to tumour while reducing the absorbed by normal tissue is key to generating an acceptable therapeutic index during any type of radiation therapy. For example, deciphering (cumulative) dose-response relationships in external beam radiation therapy (EBRT), has been subject of research. With extensive research efforts focusing on multi parametric challenges such as target delineation, focal dose-delivery (accessibility, movement, and planning) on one side and radiobiological response on the other. The latter includes tissue heterogeneity, tissue hypoxia and intrinsic tissue radiosensitivity [4]. Unfortunately, this did not convert to a means to measure dose delivery during the intervention itself but extemporaneously, so that radiobiology and dosimetry have only been able to correlate on terminal events. That was until Pogue and team successfully introduced the use of optical dosimetry via CL imaging [5]; in EBRT high energy photons generate secondary electrons via Compton scattering, which in turn can induce CL [6]. Their efforts over the last two decades indicate that CL imaging can help provide dose delivery verification in clinical care [7]. Hereby the intensity of CL at the irradiated surface helps provide a surrogate map of the dose delivered, effectively providing an on-patient-skin treatment delivery monitoring system [8]. In line with this concept Florescu and team have recently proposed to use CL imaging to support dosimetry during ^131^I thyroid therapy [9]. Their findings in hyperthyroidism and papillary thyroid carcinoma indicate that there is a direct relation between the refraction index corrected total CL intensity and dose (Gy). Hereby the number of disintegration producing CL is said to be directly proportional to *η(T*_*eff*_*2*^*−t1/Teff*^*− T*_*a*_*2*^*−t1/Ta*^*) *≃ η*T*_*eff*_*(T*_*a*_≪ *t1 (time of measurement)* ≪ *T*_*eff*_) and the total dose to *η(T*_*eff*_*− T*_*a*_*)* ≃ η*T*_*eff*_*(T*_*a*_≪ *T*_*eff*_*).* The average intake of the treatment volume is described by *η* and the parameters *T*_*eff*_ and *T*_*a*_ correspond to the loss and influx of the radioisotope into the treatment volume, respectively. Critically, literature indicates that common therapeutic radioisotopes like ^177^Lu and ^90^Y can also be detected using CL imaging ( [10]; ^90^Y (β^−^ with a maximum energy of 2.28 MeV) can produce up to 57 photons per decay in water [11]. It has even been suggested that CL imaging of β-emitting daughter nuclides supports optical detection of ^225^Ac [12].

Radionuclide therapy is a rapidly expanding oncological precision treatment method that tends to rely on fixed or empirical dosing strategies rather than optimized activity modulation [13]. Reliance on the site-specific accumulation radiopharmaceuticals to deliver targeted radiotherapy means that the degree of accumulation and the target-specificity is for a large part defined by the receptor affinity, pharmacokinetics and pharmacodynamics [14]. A process that is subjective to environmental and inter-patient variations. What is also critical to realize is that variations such as the molar activity may influence the compounds distribution profile [15]. Features that complicate radiobiological studies. In addition, radionuclide therapy is characterized by prolonged, rather low-dose-rate exposure, during which energy deposition and biological events take place simultaneously [16]. To predict absorbed-dose delivery, theranostic treatment paradigms tend to rely on pre-therapy PET (or SPECT) scans that often use the same radiopharmaceutical but then coordinated to a diagnostic radioisotope. Hereby seemingly minor differences in diagnostic and therapeutic formulation-composition and differences in the amounts of compound administered may cause discordances between diagnostic findings and therapy-delivery. Some therapeutic beta-emitting radioisotopes can also be detected directly using PET (e.g.^90^Y or ^124^I) or SPECT (e.g. ^90^Y, ^131^I, and ^177^Lu). While PET imaging helps to macroscopically assess the volumes that received an ionizing dose, it is limited by e.g. the partial volume effect. Because of that measures as the PET-based quantity SUV_max_ (a reconstruction-dependent measure) hold limited value for predicting therapeutic absorbed doses. We argue that optical dosimetry will be complementary to these efforts and may help provide a higher level of detail for relatively superficial targets [3, 17].

More detailed understanding of the radiobiology that is underlying advanced clinical theranostic treatment pathways will most likely come from the preclinic first. In the preclinic, research efforts that study innovative radioisotope treatment paradigms in mouse tumor models is widespread [18]. This coupled with the fact that widely available bioluminescence imaging devices can effectively support CL imaging, sets the stage for the exploration of optical dosimetry in theranostics. Despite suffering from signal attenuation, the preclinical scale reduction means CL should be able to provide a direct measure of the intensity of beta-interactions with e.g. tumor tissue. On top the 1–2 mm resolution will help define the gradient with which the radiation sources interact with tissues that surround the site of accumulation and inhomogeneities therein. Thereby helping provide a much-needed context for radiobiological findings. Tissue clarification techniques could help further increase the preclinical potential of CL imaging [19].

To mature theranostic strategies, it is critical to advance the dosimetric insights and to obtain a better understanding of the dose-dependent interactions that ionizing radiation has with tissue. Given its importance one would expect the field to embrace all the (potential) tools available to enhance the insight into the radiation-tissue interaction. To our surprise the concept of optical dosimetry is not gaining much traction within the nuclear medicine community. The question that arises is: Why?
